# Epidemiological characteristics and initial spatiotemporal visualisation of COVID-19 in a major city in the Middle East

**DOI:** 10.1186/s12889-021-11326-2

**Published:** 2021-07-12

**Authors:** Shahab MohammadEbrahimi, Alireza Mohammadi, Robert Bergquist, Fatemeh Dolatkhah, Mahsa Olia, Ayoub Tavakolian, Elahe Pishgar, Behzad Kiani

**Affiliations:** 1 Department of Medical Informatics, School of Medicine, Mashhad University of Medical Sciences, Mashhad, Iran; 2 Student Research Committee, Mashhad University of Medical Sciences, Mashhad, Iran; 3 Department of Geography and Urban Planning, Faculty of Social Sciences, University of Mohaghegh Ardabili, Ardabil, Iran; 4 Ingerod, Brastad, Sweden; 5 (Formerly with the UNICEF/UNDP/World Bank/WHO Special Program for Research and Training in Tropical Diseases, World Health Organization), Geneva, Switzerland; 6 Department of Microbiology and Virology, School of Medicine, Mashhad University of Medical Sciences, Mashhad, Iran; 7 Department of Anaesthesiology, School of Medicine, Shahid Sadoughi University of Medical Sciences, Yazd, Iran; 8 Department of Emergency Medicine, Faculty of Medicine, Sabzevar University of Medical Sciences, Sabzevar, Iran; 9 Department of Human Geography and Logistics, Faculty of Earth Science, Shahid Beheshti University, Tehran, Iran; 10 Department of Medical Informatics, School of Medicine, Mashhad University of Medical Sciences, Mashhad, Iran

**Keywords:** Coronavirus, COVID-19, Disease mapping, Epidemiology, Geographical information systems, SARS-CoV-2, Spatiotemporal mapping

## Abstract

**Background:**

The Severe Acute Respiratory Syndrome Coronavirus-2 (SARS-CoV-2) emerged initially in China in December 2019 causing the COVID-19 disease, which quickly spread worldwide. Iran was one of the first countries outside China to be affected in a major way and is now under the spell of a fourth wave. This study aims to investigate the epidemiological characteristics of COVID-19 cases in north-eastern Iran through mapping the spatiotemporal trend of the disease.

**Methods:**

The study comprises data of 4000 patients diagnosed by laboratory assays or clinical investigation from the beginning of the disease on Feb 14, 2020, until May 11, 2020. Epidemiological features and spatiotemporal trends of the disease in the study area were explored by classical statistical approaches and Geographic Information Systems.

**Results:**

Most common symptoms were dyspnoea (69.4%), cough (59.4%), fever (54.4%) and weakness (19.5%). Approximately 82% of those who did not survive suffered from dyspnoea. The highest Case Fatality Rate (CFR) was related to those with cardiovascular disease (27.9%) and/or diabetes (18.1%). Old age (≥60 years) was associated with an almost five-fold increased CFR. Odds Ratio (OR) showed malignancy (3.8), nervous diseases (2.2), and respiratory diseases (2.2) to be significantly associated with increased CFR with developments, such as hospitalization at the ICU (2.9) and LOS (1.1) also having high correlations. Furthermore, spatial analyses revealed a geographical pattern in terms of both incidence and mortality rates, with COVID-19 first being observed in suburban areas from where the disease swiftly spread into downtown reaching a peak between 25 February to 06 March (4 incidences per km^2^). Mortality peaked 3 weeks later after which the infection gradually decreased. Out of patients investigated by the spatiotemporal approach (*n* = 727), 205 (28.2%) did not survive and 66.8% of them were men.

**Conclusions:**

Older adults and people with severe co-morbidities were at higher risk for developing serious complications due to COVID-19. Applying spatiotemporal methods to identify the transmission trends and high-risk areas can rapidly be documented, thereby assisting policymakers in designing and implementing tailored interventions to control and prevent not only COVID-19 but also other rapidly spreading epidemics/pandemics.

## Introduction

In December 2019, a novel coronavirus, the Severe Acute Respiratory Syndrome Coronavirus-2 (SARS-CoV-2), was diagnosed in China. SARS-CoV-2 is the case of Coronavirus disease-2019 (COVID-19) that quickly spread to all countries around the world turning into a massive worldwide health concern [1, 2]. This disease commonly causes severe respiratory deficiency resulting in increased admissions into Intensive Care Units (ICUs) with high mortality rates [3, 4].

Since the outbreak of COVID-19, research has been conducted with unparalleled speed to investigate the clinical and epidemiological features of the disease. Suleyman et al. [5] conducted a study in metropolitan Detroit in the U.S. that included 463 patients with COVID-19 showing that 94% of the patients had at least one comorbidity and their most common symptoms were cough, fever and dyspnoea, respectively. Age above 60 years, male sex, pronounced obesity and chronic renal disease were soon seen as strongly associated with ICU admission and an above-average Case Fatality Rate (CFR) [5]. In Wuhan, a retrospective study performed by Zhou et al. [6] involving 191 hospitalized patients, who had been discharged or died by 31 Jan 2020, showed that 50% of them had co-morbidities; the death rate was about 28% (*n* = 54). Being old and having a higher sequential organ failure assessment score (SOFA) was significantly associated with increased CFR [6]. Another retrospective study in Wuhan, conducted on the 102 COVID-19 cases hospitalized between 31 Jan and 5 Mar 2020, reported a mortality level of 15%, with most fatalities being old [7]. Docherty et al. conducted a cohort study in the UK involving 20,133 hospital in-patients between 6 Feb and 19 Apr 2020, which showed that 60% of those admitted were men and 77% had at least one co-morbidity, while 26% of the patients (*n* = 5165) eventually died [8].

Iranian studies report similar results, e.g., a retrospective study in Tehran [9] presented the infection rate as twice as common in men as in women, a CFR of 8%, and a co-morbidity rate of 15.9% in those above 60 years. In another study conducted in Shiraz [10], 63% of the patients were male, with fatigue, cough and fever as the most common symptoms. Here, 8% overall mortality and a significant association between ICU admission and death rate, were reported [10]. Although previous studies have shown that most COVID-19 cases have a promising prognosis [11], patients with underlying chronic co-morbidities would experience more serious health consequences, including a substantial CFR [12].

This new member of the coronavirus family is highly contagious. More than one and half a year after it first emerged, as of June 10, 2021, the number of confirmed cases has reached 175 million with close to 4 million deaths globally. Of these, close to 3 million cases and more than 81,000 deaths are Iran’s share and it had the highest number of daily death caused by COVID-19 in November 2020 (> 450) [13]. In order to generate the best response strategies, analysing and representing the spatial and temporal spread of the virus is crucial for epidemiologists and health policymakers [14]. Health organizations, especially World Health Organization (WHO), have increasingly applied spatial analysis to represent and control disease outbreaks. Geographic Information Systems (GISs) have shown successful results concerning contagious diseases, and applications are been shown highly useful in mapping geographical distribution of disease prevalence as well as visualising transmission trends and modelling spatial environmental aspects of disease occurrence [15–18]. Accordingly, the GIS toolbox is highly useful for decision-making, as well as understanding the spatiotemporal dynamics and control of COVID-19 [19, 20].

Most spatial analyses of the COVID-19 outbreak have been conducted in China. For example, Tang et al. [21] reviewed the daily data flow of new cases and identified hotspots in the areas where the virus originated in the study period. Furthermore, Fan et al. [22] showed that the spatial distribution pattern of confirmed COVID-19 cases followed a particular, geographical pattern. They found hotspots to be mainly restricted to the outbreak areas, especially in densely populated areas. In another study conducted in Hubei Province of China, researchers found significant spatial autocorrelation and clustering at the local level in the study area [23]. In India, spatiotemporal analysis of confirmed COVID-19 cases at the provincial level showed significant differences in disease incidence across the Indian provinces. As well, the potential capacity of a heavy COVID-19 outbreak in India in the future, was predicted by this study [24]. In contrast, a comprehensive GIS-based study in Catalonia, Spain showed a random distribution without a clear spatial pattern and any local autocorrelation based on Global Moran’s *I* [25].

In Iran, the work of Mazar et al. [26], in one of the first studies on the spatiotemporal distribution of COVID-19 outbreaks, examined the effects of travel on the spatial distribution of the infection. Their research focused on identifying areas with high prevalence rates, especially in the provinces where the virus originated. From the GIS-based maps, it was clear that the disease spread from the north-central provinces Tehran and Qom, known as the administrative and religious centres of the country, respectively, which both are important foci for travellers [26].

In the context of spatial analysis of the COVID-19 outbreak, most previous studies have been conducted at the macro-level (world, country or province), generally without addressing the epidemiological features of the pandemic. Here, we aimed to perform a spatiotemporal analysis of new infections in a big city. To the best of our knowledge, no such study has been done in Mashhad, the second-most populous city in Iran. Besides, this study was conducted while the study area was in a vigilant status with a high epidemic alert. Our approach can be used as a basis for future spatial modelling of the disease and provide valuable knowledge for preventive measures in the study area or other similar metropolitan areas.

## Methods

### Study area and population

**Fig. 1:**
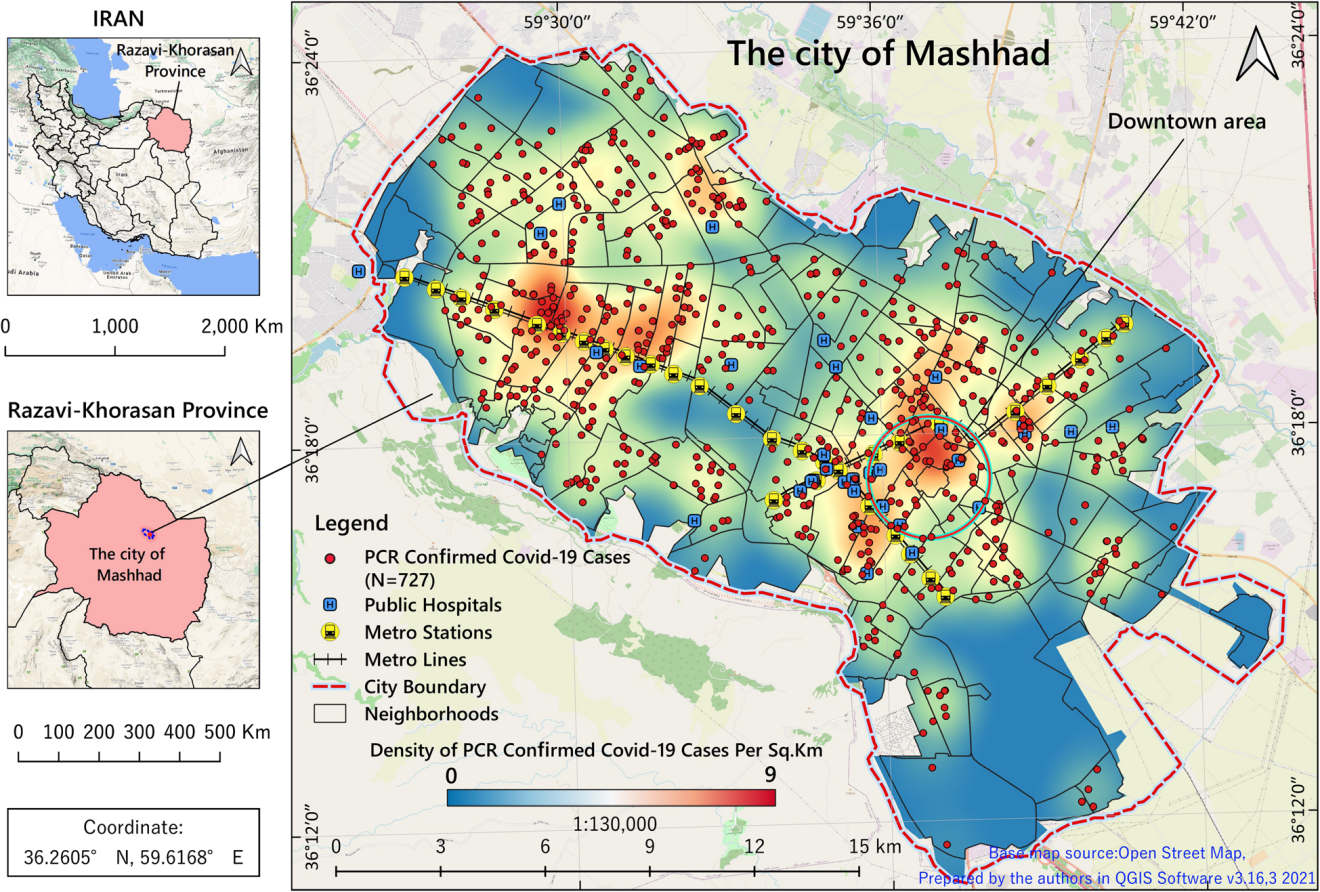
Geographical location of the study area with the distribution of hospitals and PCR-confirmed COVID-19 cases. The figure was created by the authors using QGIS free software v.3.18.3

Iran is a country in the Middle East with a population of more than 84 million people [27] located north-east of the Persian Gulf. The study area was the city of Mashhad, the capital of Khorasan-Razavi Province in north-eastern Iran. It is the most famous place of religious pilgrimage and tourist attraction in Iran attracting more than 20 million tourists and pilgrims annually [28, 29]. Mashhad is situated between latitudes 36°10’and 36°25′N and longitudes 59°25’and 59°46′E covering an area of 307 km^2^ (Fig. 1). According to the 2016 national census, the city population was 3,372,660 [30]. Mashhad consists of about 17 municipality regions, 175 districts and 1301 census tracts. In our study, we used census tracts as the finest scale of analysis to make the results more accurate and more detailed.

### Study design and data collection

This retrospective study was conducted at two levels. At the first level, all collected data were subjected to a statistical analysis rendering the general epidemiological characteristics of the pandemic, while at the second, we focused on a space-time analysis to identify the geographical trends of dissemination and clustering patterns of the disease.

The basis of the study was the collected COVID-19 data from the Mashhad University of Medical Sciences (MUMS). The study covered 3 months with the start coinciding with the beginning of the COVID-19 outbreak in Mashhad (14 Feb 2020) and continued until 11 May 2020. The data included 4000 people referred to the health-care centres and hospitals due to COVID-19 infection with cases either confirmed clinically or by laboratory tests. For unequivocal confirmation of this infection, a Reverse Transcription Polymerase Chain Reaction (RT-PCR) test is required [31]. The demographic and clinical database included patients’ residence address, sex, age, date of symptoms onset, Length of Stay (LOS) in the hospital, underlying diseases, common symptoms, exposure history, disease severity and disease outcome.

### Statistical analysis

At the first level, continuous variables were expressed as median and Inter Quartile Range (IQR) and testing the null hypothesis using the Mann-Whitney U test. We used median and IQR because the data did not follow a normal distribution. Categorical variables were expressed as number and percentage and compared by Fisher’s exact test (*χ2*) between men and women as well as between survivors and non-survivors groups. To determine the effect of age, sex, ICU admission, LOS and co-morbidities on mortality, univariate logistic regression was used. We also applied the un-adjusted Odds Ratio (OR) with a 95% Confidence Interval (CI). To do this, 2,943 patients with a certified outcome (death or recovery) were included. All tests were two-sided with *p* < 0.05 considered statistically significant. All statistical analyses were performed using R software, version 4.0.5 (R Foundation for Statistical Computing) and Microsoft Excel 2016.

### Spatiotemporal analyses and mapping techniques

For this study, we only used a subdivision comprising those COVID-19 cases in Mashhad City that had been confirmed by RT-PCR (*n* = 727). Any data outside the city of Mashhad were excluded from the spatiotemporal approach. With the study area located around the 36°N and 59°E, the WGS_1984_UTM_Zone_40N projection system was used for projecting the GIS layer. Five age groups including 0–14, 15–24, 25–44, 44–64, and ≥ 65 years old were used to calculate the age- and sex-adjusted incidence and mortality rates of the COVID-19 in each census tract. ArcGIS software, version 10.7.1 (ESRI, Redlands, CA, USA) was used for the spatial analysis.

#### Kernel density estimation (KDE)

KDE, one of the non-parametric and distance-based techniques for analysing the spatial intensity of point occurrences, shows the locations with the highest or lowest density on a smooth density surface map. Here, the value of each cell at the raster (image file format) surface refers to the number of incidence events. In this study, we used a 30-m cell size within a 2500-m bandwidth displaying a smoothed spatial density map [32].

#### *Hotspot* analysis (Getis-Ord Gi*)

A hotspot is an area within a prescribed limit with concentration or dispersion of occurrences of the same value [33, 34]. The Getis-Ord Gi* statistics detects the presence of local spatial autocorrelations, i.e., hotspots, with calculations based on Z-score and *p*-value. We applied this statistic to identify COVID-19 hotspots and coldspots based on age- and sex-adjusted incidence rates at the census tract level as spatial features. The index is computed by the following equation:

1 $$ {G}_i^{\ast }=\frac{\sum \limits_{j=1}^n{w}_{i,j}{X}_j\overline{X}-\sum \limits_{j=1}^n{w}_{i,j}}{S\sqrt{\frac{\left[n\sum \limits_{j=1}^n{w}_{i,j}^2-{\left(\sum \limits_{j=1}^n{w}_{i,j}\right)}^2\right]}{n-1}}} $$

Where X_*j*_ is the attribute value, COVID-19 incidence in this study, for feature j; w_*ij*_ is the spatial weight between feature *i* and *j*; n is equal to the total number of features; $\overline{X}$ is the mean of the corresponding attribute and *S* is the standard deviation of X_*j*_. The G_i_* statistic is a z-score, so no further calculations are required. Selecting the proper conceptualization relationships method is important to measure the G_i_* statistic. Among different methods of spatial conceptualization relationships, in this study, the Contiguity Edges Corners method was used for all calculations. This method is the most appropriate and effective method for polygonal features [35].

#### Spatial autocorrelation

There are various methods for measuring spatial autocorrelation. The global methods are more sensitive to departures from the null hypothesis (random distribution) but do not tell where the clusters are, which is possible when applying local methods. We used Global Moran’s Index (GMI) [36] and Anselin’s Local Moran’s Index (ALMI) since they are generally more accurate concerning measuring autocorrelation than other statistics [36]. The value of GMI and ALMI vary between + 1 and − 1. The ALMI can detect four types of clusters: High-High (HH) reflecting high incidence areas surrounded by other high incidence areas, High-Low (HL) high incidence areas surrounded by low incidence areas, Low-Low (LL) low incidence areas surrounded by other low incidence areas, and Low-High (LH) low incidence areas surrounded by high incidence areas [37].

### End-points

The end-point was in principle survival after infection and discharge from the hospital. We investigated this with regard to ICU admission, use of ventilators and coma taking into account gender, age, co-morbidities, symptoms and severity of the infection and the number of days counted from hospital admission to a certified outcome.

## Results

### Statistical analysis

Of the 4000 patients included in this study, 2352 (58.8%) were males and 1648 (41.2%) females. Table 1 shows a comprehensive comparison of the genders with respect to their responses to the infection, while Table 2 is focused on the health outcomes in those with evident health outcomes, i.e. survivors (*n* = 2236) and non-survivors (*n* = 707). We excluded all those continuing to receive care at the date of reporting (*n* = 1057) as the outcome was then unknown.

**Table 1:** Baseline characteristics by gender of all COVID-19 cases referred to healthcare centres in Mashhad City from Feb 14, to May 11, 2020

Reference	All patients	Males	Females	*p*-value
Number of patients (%)	4000 (100)	2352 (58.8)	1648 (41.2)	
Median age of the patients	57.0 years	56 .0 years	58 .0 years	0.0104*
Age range	39.0–71.0	39.0–70.0	39.0–73.0	
Days from onset to hospital admission	2.0	2.0	2.0	0.9783
Range of days	1.0–4.0	1.0–4.0	0.0–4.0	
Days from hospital admission to outcome	5.0	5.0	5.0	0.4751
Range of days	2.0–8.0	2.0–8.0	2.0–8.0	
**Demographics & clinical characteristics**	**Number (%)**	**Number (%)**	**Number (%)**	
Co-morbidity				
Diabetes	491 (12.27)	252 (10.71)	239 (14.50)	0.0003*
CVD	805 (20.12)	404 (17.2)	401 (24.3)	< 0.0001*
Liver disorders	112 (2.80)	61 (2.59)	51 (3.09)	0.3444
CRD	155 (3.87)	95 (4.04)	60 (3.64)	0.5206
Nervous diseases	99 (2.47)	64 (2.72)	35 (2.12)	0.2314
COPD	236 (5.90)	115 (4.89)	121 (7.34)	0.0011*
Malignancy (any)	99 (2.47)	59 (2.50)	40 (2.42)	0.8706
Initial symptoms				
Fever	2177 (54.42)	1308 (55.61)	869 (52.73)	0.0716
Cough	2377 (59.42)	1383 (58.80)	994 (60.31)	0.337
Dyspnoea	2777 (69.42)	1606 (68.28)	1171 (71.05)	0.0609
Weakness	778 (19.45)	452 (19.21)	326 (19.78)	0.6574
Myalgia	574 (14.35)	331 (14.07)	243 (14.74)	0.5507
Dizziness	338 (8.45)	206 (8.75)	132 (8.0)	0.4020
Sore throat	434 (10.85)	262 (11.13)	172 (10.43)	0.4819
Sputum	74 (1.85)	37 (1.57)	37 (2.24)	0.1206
Diarrhoea	137 (3.42)	79 (3.35)	58 (3.51)	0.7834
Nausea or vomiting	332 (8.30)	182 (7.74)	150 (9.10)	0.1238
Headache	325 (8.12)	184 (7.82)	141 (8.55)	0.4038
Chest pain	318 (7.95)	181 (7.70)	137 (8.30)	0.4773
Abdominal pain	70 (1.75)	32 (1.36)	38 (2.30)	0.0248*
Arthralgia	235 (5.87)	133 (5.65)	102 (6.19)	0.4792
Pharyngitis	49 (1.22)	27 (1.14)	22 (1.33)	0.5967
Conjunctivitis	30 (0.75)	17 (0.72)	13 (0.78)	0.8117
Abnormal chest X-ray	1059 (26.47)	617 (26.23)	442 (26.82)	0.6786
End-points				
ICU admission	502 (12.55)	307 (13.05)	195 (11.83)	0.2516
Ventilator	772 (19.30)	449 (19.09)	323 (19.59)	0.6878
Coma	24 (0.60)	16 (0.68)	8 (0.48)	0.4322
Exposure history (last 14 days)				
Travel	83 (2.07)	58 (2.46)	25 (1.51)	0.0382*
Exposed at medical centres	323 (8.07)	185 (7.86)	138 (8.37)	0.5615
Exposed to possibly infected individuals	267 (6.68)	155 (6.59)	112 (6.79)	0.7973
Exposed to animals	175 (4.37)	118 (5.02)	57 (3.46)	0.0177*
Being healthcare staff	281 (7.03)	158 (6.71)	123 (7.46)	0.3636
Disease severity	…	…	…	0.0404*
General	3228 (80.70)	1927 (81.93)	1301 (78.94)	…
Severe	748 (18.70)	414 (17.60)	334 (20.27)	…
Critical	24 (0.60)	11 (0.47)	13 (0.79)	…
Disease outcome	…	…	…	0.0437*
Non-survivor	707 (17.67)	430 (18.3)	277 (16.8)	…
Survivor	2236 (55.90)	1334 (56.7)	902 (54.7)	…
Ongoing care	1057 (26.43)	588 (25.0)	469 (28.5)	…
COVID-19 confirmation	…	…	…	0.0118*
PCR confirmation	1325 (33.1)	816 (34.7)	509 (30.9)	…
Clinical confirmation	2675 (66.9)	1536 (65.3)	1139 (69.1)	…

*CVD* Cardiovascular Diseases, *CRD* Chronic Renal Diseases, *COPD* Chronic Obstructive Pulmonary Diseases, *ICU* Intensive Care Unit. *Significant values

**Table 2:** Baseline characteristics stratified by mortality and survival of patients with a certified outcome in Mashhad City from Feb 14, to May 11, 2020

Reference	All patients	Non-survivors	Survivors	*p*-value
Number of patients (%)	2943 (100)	707 (24)	2236 (76)	
Median age of the patients	57.0	68.0	52.0	0.0001*
Age range	40.0–71.0	59.0–79.0	36.0–66.0	
Days from onset to hospital admission	2.0	2.0	2.0	0.0695
Range of days	1.0–4.0	0.0–4.0	1.0–4.0	
Days from hospital admission to outcome	5.0	6.0	4.0	< 0.0001*
Range of days	2.0–8.0	2.0–10.0	2.0–7.0	
**Demographics & Clinical Characteristics**	**Number (%)**	**Number (%)**	**Number (%)**	
Sex	…	…	…	0.5832
Female	1179 (40.06)	277 (39.18)	902 (40.34)	…
Male	1764 (59.94)	430 (60.82)	1334 (59.66)	…
Co-morbidity				
Diabetes	405 (13.76)	129 (18.25)	276 (12.34)	0.0001*
CVD	614 (20.86)	197 (27.86)	417 (18.64)	< 0.0001*
Liver disorders	89 (3.02)	28 (3.96)	61 (2.72)	0.0953
CRD	120 (4.07)	38 (5.37)	82 (3.67)	0.0453*
Nervous diseases	69 (2.34)	28 (3.96)	41(1.83)	0.0011*
COPD	181 (6.15)	71 (10.04)	110 (4.91)	< 0.0001*
Malignancy (any)	73 (2.48)	39 (5.51)	34 (1.52)	< 0.0001*
Initial symptoms				
Fever	1720 (58.44)	385 (54.45)	1335 (59.70)	0.0135*
Cough	1874 (63.67)	378 (53.46)	1496 (66.90)	< 0.0001*
Dyspnoea	2111 (71.72)	579 (81.90)	1532 (68.51)	< 0.0001*
Weakness	533 (18.11)	122 (17.25)	411 (18.38)	0.4984
Myalgia	429 (14.57)	71 (10.04)	358 (16.01)	< 0.0001*
Dizziness	247 (8.39)	80 (11.31)	167 (7.47)	0.0013*
Sore throat	352 (11.96)	47 (6.64)	305 (13.64)	< 0.0001*
Sputum	55 (1.86)	5 (0.70)	50 (2.23)	0.0088*
Diarrhoea	101 (3.43)	16 (2.26)	85 (3.80)	0.0501
Nausea or vomiting	246 (8.36)	39 (5.51)	207 (9.26)	0.0017*
Headache	245 (8.32)	35 (4.95)	210 (9.39)	0.0001*
Chest pain	223 (7.57)	49 (6.93)	174 (7.78)	0.2906
Abdominal pain	39 (1.32)	3 (0.42)	36 (1.61)	0.0162*
Arthralgia	176 (5.98)	25 (3.53)	151 (6.75)	0.0016*
Pharyngitis	37 (1.25)	6 (0.85)	31 (0.14)	0.2633
Conjunctivitis	18 (0.61)	2 (0.28)	15 (0.72)	0.1984
Seizure	22 (0.75)	6 (0.85)	16 (0.71)	0.7203
Abnormal chest X-ray	697 (23.68)	186 (26.30)	511 (22.85)	0.0596
End-points				
ICU admission	338 (11.48)	150 (21.21)	188 (8.40)	< 0.0001*
Ventilator	549 (18.65)	209 (29.56)	340 (15.20)	< 0.0001*
Coma	19 (0.65)	9 (1.27)	10 (0.45)	0.0168*
Disease severity status	…	…	…	0.0033*
General	2418 (82.16)	551 (77.93)	1867 (83.50)	…
Severe	503 (17.09)	150 (21.22)	353 (15.78)	…
Critical	22 (0.75)	6 (0.85)	16 (0.72)	…

*CVD* Cardiovascular Diseases, *CRD* Chronic Renal Diseases, *COPD* Chronic Obstructive Pulmonary Diseases, *ICU* Intensive Care Unit. *Significant values

#### Age and sex

The median age of all patients was 57 years (IQR [39-71]). Seven percent (280 cases) were less than 20 years old and 43% (1720) older than 60 years. Women were slightly older than men (58 vs. 56 years of age) and had no significant differences in terms of common symptoms. However, as seen in Table 1, they had more often co-morbidities, such as Cardiovascular Diseases (CVD) (24.3% vs. 17.2%), diabetes (14.5% vs. 10.7%) and Chronic Obstructive Pulmonary Diseases (COPD) (7.3% vs. 4.9%), (*p* < 0.05). More men (*n* = 2352, 58.8%) than women (*n* = 1648, 41.2%) were admitted because of COVID-19 symptoms to health centres and hospitals. Especially among the non-survivors, the ratio of men to women was 60.8% (*n* = 430) to 39.2% (*n* = 277).

#### Symptoms

**Fig. 2:**
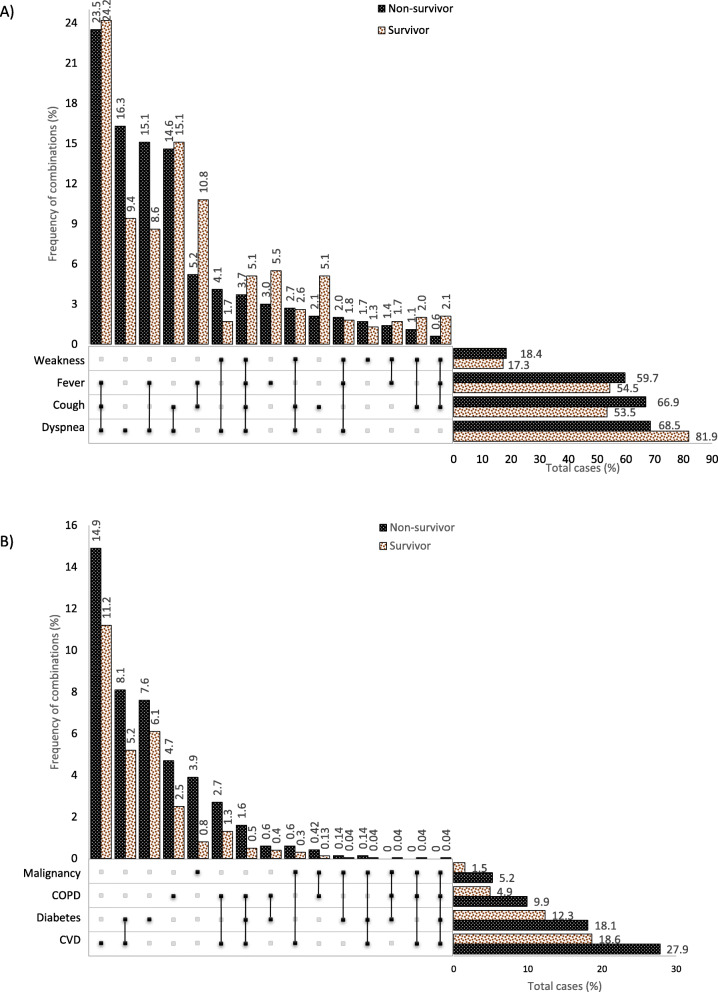
Combinations of COVID-19 symptoms (**A**) and co-morbidities (**B**) in patients with various outcomes (non-survivors vs. survivors)

In this study, 17 types of COVID-19 symptoms were reported. The most common were dyspnoea (69.4%), cough (59.4%), fever (54.4%) and weakness (19.45%). In order to clarify the prevalence of pre-existing medical conditions, we created combination charts (Fig. 2) that show the prevalence of symptoms and co-morbidities alone or combined. Figure 2a shows the rate of the four most common symptoms and various combinations in relation to survivors and non-survivors (*n* = 2943). As can be seen, approximately 82% of those who did not survive, suffered from dyspnoea, and 16.5% of them experienced this symptom without any combination with other symptoms. The most common combination of symptoms was the triple group of dyspnoea-cough-fever (23.5% mortality vs. 24.2% survival), followed by the dual combinations of dyspnoea-fever (15.1% vs. 8.6%), dyspnoea-cough (14.6, 15.1%) and cough-fever (5.2, 10.8%). Only 0.6% of non-survivors and 2.5% of survivors had experienced the combination of cough-fever-weakness which it means having these symptoms without suffering from shortness of breath was really rare.

#### Co-morbidities

Among the seven major underlying diseases, CVD (20.1%), diabetes (12.3%), COPD (5.9%) and malignancies (2.5%) were the most common in COVID-19 patients. All of those with underlying diseases, except liver disorders (*p* = 0.0953), showed higher rates in the non-surviving group vs. the surviving one (*p* < 0.05) (Table 2). Also, as shown in Fig. 2b, where the co-morbidities and their combinations are presented in relation to survival. In this figure, the highest CFR was related to those who suffered from CVD (27.9%) or diabetes (18.1%) (including the combinations of these two co-morbidities). Out of those in the CFR group who had only one co-morbidity, CVD was the most common (14.9%) followed by diabetes (7.6%), COPD (4.7%) and malignancy (3.9%). The highest degree of the combined co-morbidities was CVD-diabetes (8.1% mortality vs. 5.2% survival) and CVD-COPD (2.7% mortality vs. 1.3% survival). In the category of three underlying diseases, the triple of CVD-diabetes-COPD had the highest mortality rate (1.6%). Only one person (a male aged 72 years) experienced all four underlying diseases; against all odds, he survived.

#### Patient outcomes

**Fig. 3:**
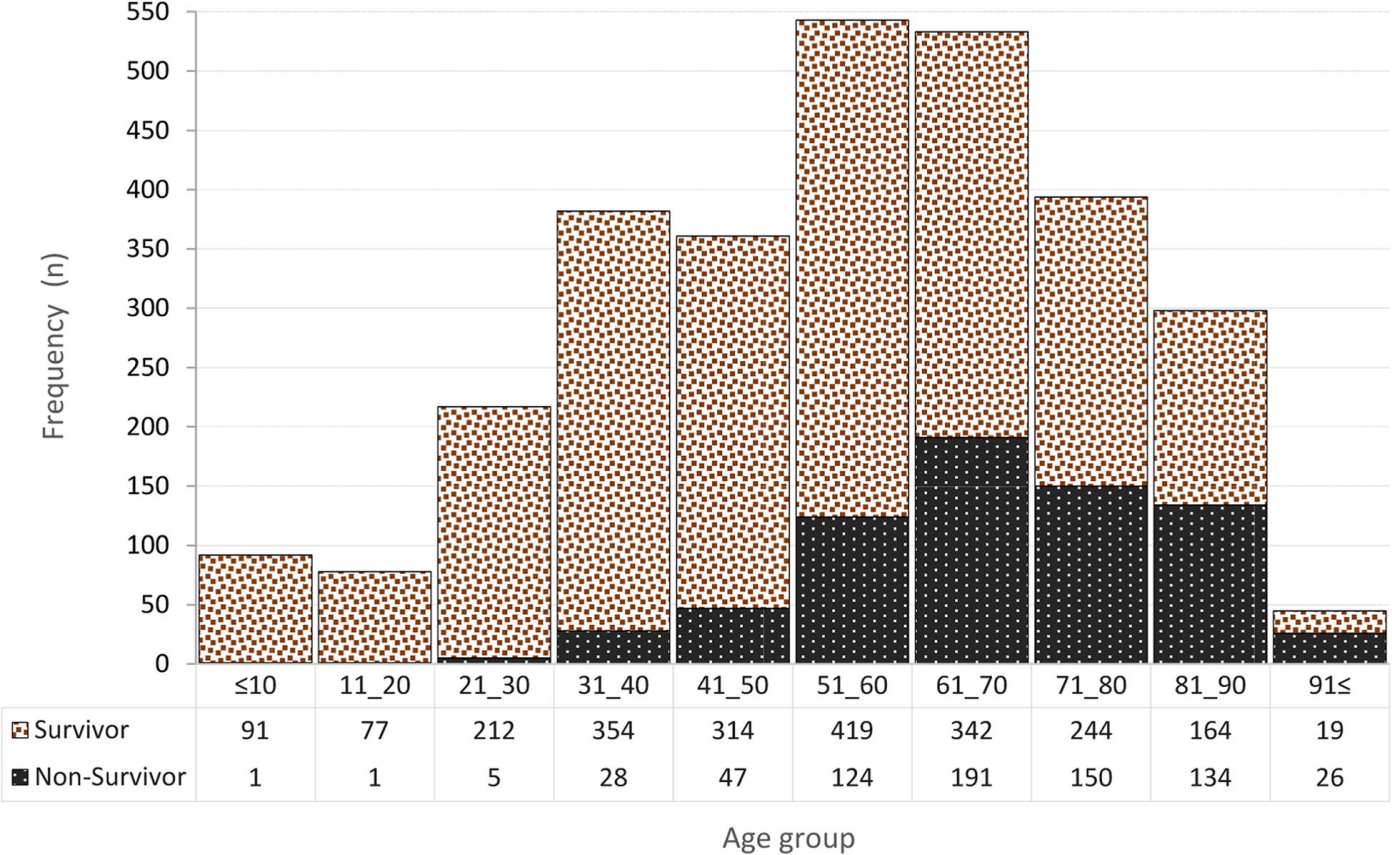
Distribution of COVID-19 cases and deaths in ten age groups

The three types of outcomes in our data are telling. Overall, almost 56% (*n* = 2236) of the patients were discharged in good condition, 17.7% (*n* = 707) died and 26.5% (*n* = 1057) were under continued care and thus deemed to have an equivocal outcome at the date of reporting (Table 1). Importantly, as seen in Table 2, the median age in the non-survivor group (68, IQR [59–79]) was 16 years above that of the survivors (52, IQR [36-66]), (*p* < 0.0001). By categorising the age into 10 categories (10-year intervals), as shown in Fig. 3, only 1 % of all deaths (7/707), occurred among the first three age groups (≤10, 11–20 and 21–30 years of age). The highest frequency of COVID-19 infection was observed in the 51–60 age group (18.5% (543/2943)). The chart shows clearly that the CFR rose with age in each of the four oldest age groups (61–70, 71–80, 81–90 and > 90 years) by reaching 35.8% (191/533), 38.0% (150/394), 44.9% (134/298) and 57.8% (26/45), respectively. It can thus be said that, in the current study, 71% (501/707) of all CFRs caused by COVID-19 occurred in ages over 60 years.

#### End-points

Based on Table 2, more than 60% of the non-survivors were men. The use of ventilators for non-survivors (29.5%) was almost twice that given for the survivors (15.2%), and ICU admission was more than twice for those who died (21.2%) compared to that for the survivors (8.4%), both with a highly significant *p*-value (*p* < 0.0001). Also, the number of people who experienced coma was higher in the non-survivor group (*p* = 0.0168). The median number of days counted from hospital admission to death or discharge was significantly different between the people who died (6 days, IQR [2-10]) and those who survived (4 days, IQR [2-7]). However, there was no significant difference between the time from symptom onset to hospital admission between these groups (*p* = 0.0695).

#### History of exposure

Of all cases, 7% (*n* = 281) were healthcare staff, three of whom (0.5%) died. The rate of exposure at medical centres, by meeting possibly infected individuals and/or animals were 8, 7 and 4.3%, respectively. Among the infected cases, men had travelled significantly more than women (*p* < 0.05).

#### Admission trend

**Fig. 4:**
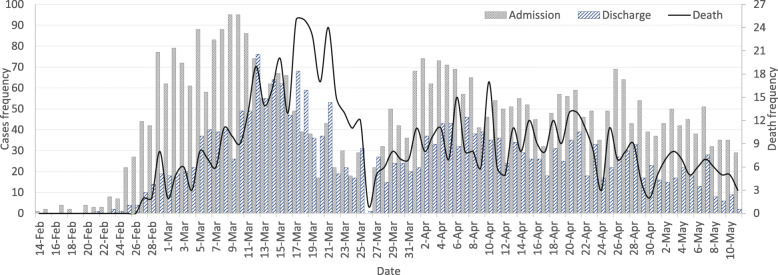
Trends of admission, mortality, and discharge rates of COVID-19 cases in the study period

As shown in Fig. 4, covering 3 months of hospital admissions concerning COVID-19 in the city of Mashhad, each admission peak was followed by a pair of discharge and mortality peaks, the former about three times higher than the latter.

#### Risk factor analysis

**Fig. 5:**
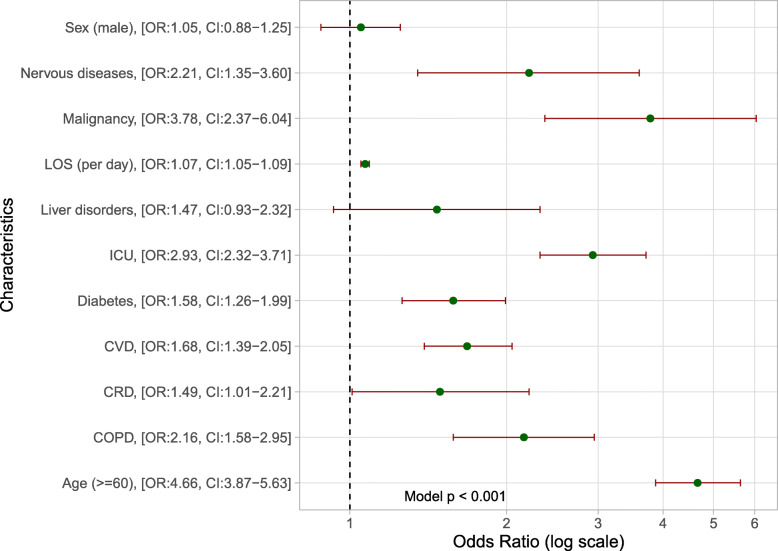
Odds Ratio (OR) of death in COVID-19 patients with a certified outcome

By including the COVID-19 variables in univariate logistic regression, we found that older age (≥60 years) was associated with increased odds of death (Fig. 5). People belonging to this category had almost five times the vulnerability in this regard (OR: 4.7, CI [3.8–5.6]) compared to those under 60 years. Patients with malignancy (OR: 3.8, CI [2.4–6.0]), diseases of the nervous system (OR: 2.2, CI [1.4–3.6]) or COPD (OR: 2.2, CI [1.6–3.0]) also had higher odds of death. The univariate regression also revealed these odds increased along with the time of hospitalization on a daily basis (OR: 1.07, CI [1.05–1.09]). ICU admission was seen as another risk factor as it was associated with a threefold increase in the odds of death (OR: 2.9 CI [2.3–3.7]).

### Spatiotemporal analysis

#### Disease spatiotemporal trends

**Fig. 6:**
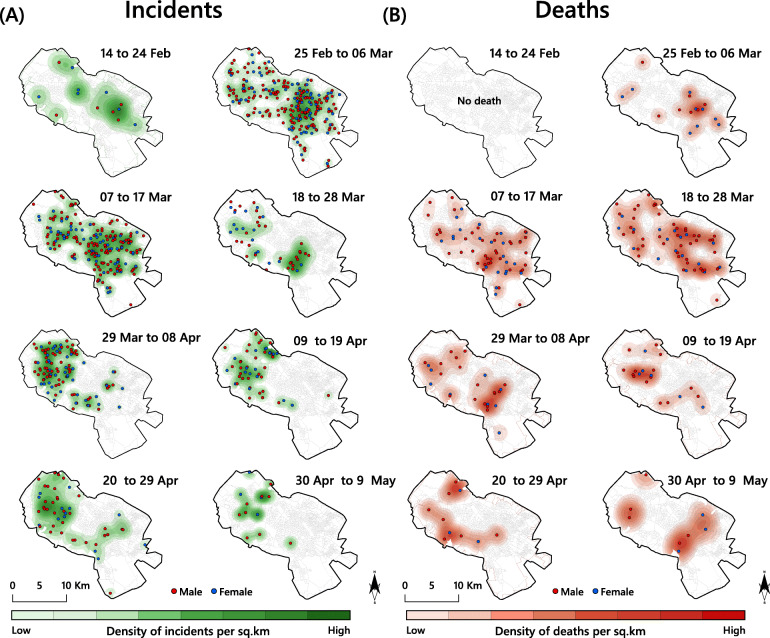
Spatiotemporal patterns of confirmed COVID-19 cases, across eight time periods (**A**) Incidence; (**B**), Mortality (expressed as the number of cases per km^2^). The figure was created by the authors using ArcGIS v.10.8. A correct license was attributed by the authors

Figure 6 shows the spatiotemporal progression of the COVID-19 outbreak across eight 10-day periods (14 Feb to 9 May, 2020) based on the KDE results with the strength of incidence and mortality rates displayed by colour code. As can be seen in Fig. 6a, the infection spread within 3 weeks from a general low presence (*n* = 13) in the third week of February to a peak with a strong focus in the downtown area in the south-eastern part of the city that was maintained for 3 weeks with the highest density (4 events per km^2^, *n* = 252) around the end of the first week of March. Then the infection waves first subsided but soon resumed forming another focus in the north-western part of the city that held sway between 20 Mar and 8 Apr after which it gradually disappeared. Figure 6b tell a different story; while there were no deaths in the first period, the second has a close resemblance with the first period of disease incidence Fig. 6a, possibly indicating that time between the onset of symptoms lasted between 1 and 2 weeks; on the other hand, we do not know if the events shown refer to the same persons. Still, the temporal lag is quite evident with mortality due to COVID-19 beginning on Feb 25, peaking between Mar 18 and 28 before subsiding. It is noteworthy that, the incidence and mortality of the infection gradually decreased in the downtown area from this date. Out of all investigated patients in the study area (*n* = 727), 205 (28.2%) did not survive and 66.8% of these cases were men. Finally, it can be stated that apart from the time progression no other parallel significant difference was found in terms of incidence and/or mortality in different parts of the city. What is clear is that the mortality in the central and western parts of the city was higher than in other areas.

#### Age- and sex-adjusted rates

**Fig. 7:**
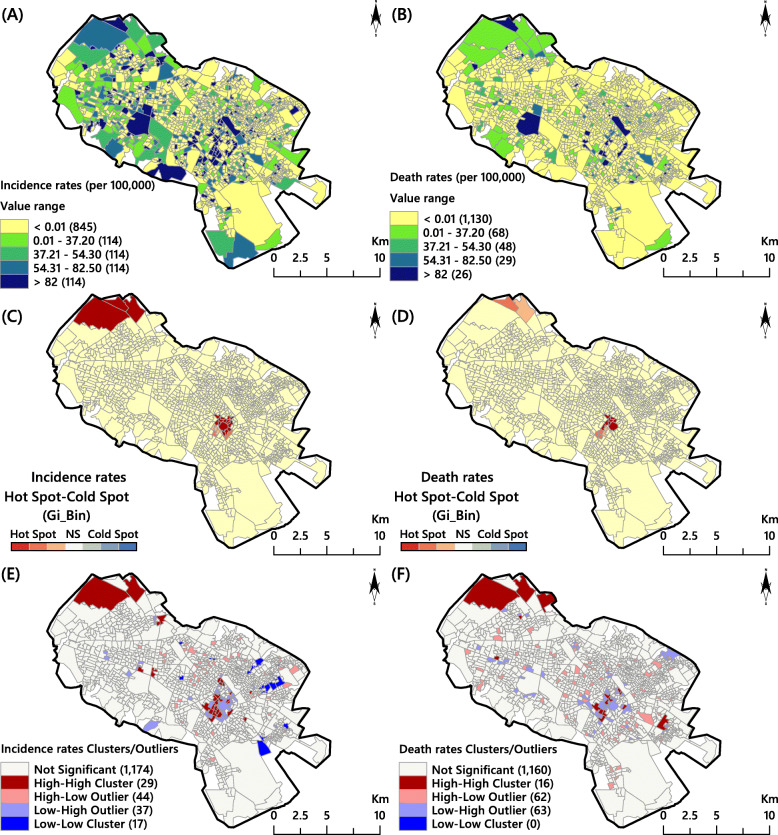
The maps of COVID-19 spatial analysis results: **A**-Incidence rates, **B**-Death rates, **C**-Incidence Hot-spots, **D**- Death Hot-spots, **E**-Incidence Cluster/Outlier, **F**- Death Cluster/Outlier. The figure was created by the authors using ArcGIS v.10.8. A correct license was attributed by the authors

Figure 7a shows the sex- and age-adjusted incidence rates map (per 100,000 persons). According to this, the census tracts in the Central Business District (CBD), the western and the north-western parts of the city (17.5%) experienced a high incidence (> 54.31 per 100,000 persons). Figure 7b, which presents the spatial pattern of mortality, showed a similar picture, but only 4.2% of the census tracts experienced high number of deaths

#### Hotspot analysis

The outcome of the Getis Ord Gi* statistic is shown in Fig. 7c. Here, 24 census tracts in the north-western and downtown parts of the city showed COVID-19 hotspots (with 99, 95, and 90% confidence). Regarding the spatial pattern of mortality, as shown in Fig. 7d, 15 census tracts formed hotspots. The north-western region can be described as a combination of industrial and residential land use close to rural areas with low population density. The CBD area was extremely significant with respect to risk (hotspot point with 99% confidence), which can be due to commercial and pilgrimage situations.

#### Cluster and outlier analysis

The GMI results for incidence and mortality were 0.072 (*z* = 5.38, *p* = 0.001), and 0.027 (*z* = 3.85, *p* = 0.001), respectively, which indicated that the COVID-19 distribution was more spatially clustered than expected if the underlying spatial processes were random (*p* = 0.001). In other words, high-risk areas were surrounded by neighbours with high incidence risk. In order to determine how the incidence and mortality rates were clustered in the city, the ALMI was calculated. According to Fig. 7e, there was a bit of spatial autocorrelation of the COVID-19 epidemic incidence among the 1174 census tracts. In terms of incidence rates, we identified H-H clusters (29 tracts), mainly in the central and north-western parts of the city which showed spatial autocorrelation (*p* < 0.05). Only one census tract to the west in the city had an H-H cluster. However, the L-L clusters (17 tracts) showed a spatial autocorrelation in the eastern regions (*p* < 0.05), while the H-L and L-H outliers were dispersed throughout the city. According to Fig. 7f, there were no spatial COVID-19 mortality clusters among the 1160 census tracts (*p* > 0.05). In addition, the clustered mortality pattern differed substantially from that of the incidence rates since the L-L death rate values were statistically not significant and not clustered. But spatially, just as the incidence rates, the central and north-western areas were clustered with high values (*p* < 0.05). In both the incidence and mortality maps, the H-L outliers were located next to each other in downtown (*p* < 0.05).

## Discussion

To the best of our knowledge, this is the first retrospective study conducted in Mashhad City with a simultaneous focus on statistical and spatiotemporal analysis. Our results show a mortality rate of 17.7% and the median age of patients was higher than that found in Chinese and Australian studies [37, 71], but lower compared to American and European reports [8, 38]. In accordance with most other studies [8, 39–41], infected women were found to be slightly older than men in our study (2 years on average). We also found the most frequent COVID-19 infections (36.6%) occurring in the 51–60 and 61–70 age groups (Fig.3), findings closely in line with studies conducted in Italy and China [42, 43]. However, in contrast to studies in other countries [8, 38, 44], the frequency of infection in younger age groups (31–40 and 41–50) in our study was considerable (25.2%), a fact that could be due to the lower median age of the Iranian population, which means that a larger proportion of younger people in Iran compared to many other countries are exposed in their daily activities [45]. Following the studies comparing the characteristics of survivors and non-survivors of COVID-19 patients [6, 7, 46, 47], we also found those who did not survive were on average much older than those who did. The CFR in elderly patients (age ≥ 60) investigated by OR was almost five times more than the younger patients, indicating that senility might be a risk factor for mortality. This is hardly surprising as older patients are more vulnerable to serious complications due to a less active immune system and, particularly because they commonly have various underlying diseases. In order to predict poor prognosis in these patients, Ma et al.’s [48] suggested frailty assessment in the early stage of disease, which could help to identify potentially severe pneumonia.

The findings related to pre-existing medical conditions are in line with Emami et al.’s study [45], which reviewed the prevalence of underlying diseases in COVID-19 cases. As seen in Table 2, all investigated comorbidities, except liver disorders, were significantly higher more common in non-survivors compared to those who survived (*p* < 0.05). Although most studies [6, 46, 47] show a higher prevalence of underlying diseases in the deceased patients, all did not find any significant differences regarding the prevalence of co-morbidities in non-survivors compared to survivors [7, 10]. However, although there is a high variability of the prevalence rates for CVD (7.5–40.0%) and diabetes (8.0–38.0%), it is well-known that these maladies are the most prevalent pre-existing medical conditions among COVID-19 patients [5, 6, 8, 10, 11, 39, 43, 46, 49]. As shown on the upper side of Fig. 2b, CVD was unaccompanied with any other co-morbidity in nearly 15% of those who died in Mashhad, which indicates that CVD is a serious condition that potentially can lead to the poor prognosis of COVID-19 [50, 51]. The suggestion that can be made considering this high risk for CVD patients is the use of tele-rehabilitation, which has been well proven in previous studies [52, 53]. In agreement with our results, a nationwide analysis conducted in England has shown that diabetes (both types) is associated with a significantly increased CFR by COVID-19 [54]. Our results revealed that patients with malignancies also have a much higher risk, which increases the CFR almost four times. Immunosuppressive therapies and intrinsic frailty are possibilities that potentially put this population at risk [46].

According to the results of a meta-analysis [55] and the report from the Centres for Disease Control and Prevention (CDC) in the US [56], fever, cough, dyspnoea and fatigue are the most commonly manifested symptoms in COVID-19 patients. Regardless of the order, our findings agree with this. We found a significant difference in the frequency of main manifested symptoms between the deceased and the survivors (Table 2), this difference was not significant in other studies [6, 47]. Fever has been reported as the most common symptom in most studies [5, 6, 11, 12, 39, 57], while others, similar to our results (70%), report dyspnoea as the most common symptom [47, 58, 59]. As shown, the combination of dyspnoea-cough-fever was the most commonly manifested symptom among the patients who did not survive (23.5%) (Fig. 2a). Since 82% of deceased patients had dyspnoea as one of the manifested symptoms and also 16.5% of them reported it as the only symptom of disease, it can be claimed that suspicious cases presenting with this symptom should be taken seriously even if it is the sole symptom.

In agreement with the results of other studies [6, 7, 9–11, 46], our results showed that COVID-19 infects males more often than females (Table 1). However, contrary to our finding that the CFR in the male sex was not significantly higher than that in the female (OR: 1.05, CI [0.88–1.25], a majority of studies maintain that sex may influence mortality due to COVID-19 [5, 8, 9]. This is an important finding that must be further studied.

In contrast to reports in influential journals [8, 38] claiming that mechanical respiration results in very high CFRs, our study found only a 38% (209/549) mortality in patients receiving mechanical ventilation due to COVID-19 induced shortness of breath. However, we found admission to an ICU as a risk factor that increased the CFR up to three times. The median duration of symptoms manifestation before admission was 2 days, i.e. slightly lower than that of English patients (4 days, IQR [1-8]) and much lower than that of Chinese patients (11 days, IQR [8–14]) [6, 8]. The 5-day median LOS in our study agrees quite well with that by Richardson et al. [38] (4 days, IQR [2-7]), but it was less than half of that in Bhatraju et al.’s study [58] (12 days, IQR [8-18]). It is noteworthy that, based on the results of regression analysis, increased LOS values are associated with higher CFRs. The relatively low LOS rate in our study may be due to the sudden increase of infected patients leading to a shortage of medical equipment and active hospital beds at the early stages of the COVID-19 pandemic in Iran.

Lockdown of cities can be a great solution to control and prevent the spread of the disease [60–62]. In Iran, the lack of public awareness promoted the rapid spread of COVID-19 and the rate of hospital admission and mortality increased dramatically. However, by raising public awareness about the seriousness of the COVID-19 threat, imposing travel restrictions and closing schools and universities as mentioned by other studies [63, 64] as an effective factor in reducing the burden of COVID-19, the first peak of the outbreak was eventually largely controlled (Fig. 4). But perhaps the most important factor in this control was the annual Iranian Nowruz two-week holiday. Starting on March 20, it turned out to be a useful excuse for a national lockdown, e.g., already on March 14, 1 week before the holiday, the largest commercial and pilgrimage centre in the CBD of the city was closed due to the outbreak of COVID-19. This shows that a timely urban policy can be very effective. But due to the economic fragility of the Iranian community, the closure of the central parts of the cities cannot be sustained for the longer term. Due to the complex nature of the COVID-19, short-term temporal and spatial policies can fail. Hence, more efficient space-time policies are needed.

Using hotspot analysis, we identified statistically significant transmission areas in terms of the spatial autocorrelation of COVID-19 incidence and deaths in the two prominent areas of the city characterized by high traffic and interchange, i.e. the central part of the city or CBD and the industrial area in north-western Mashhad. The number and density of pilgrimage, commercial, and tourist services centres (as well as hotels and inns) are high in the former, while the latter consists of industrial areas surrounded by rural areas from where large numbers of people commute to work. In addition, as concluded in Mazar et al.’s study [26], in the frequently visited cities where travel back and forth is high and permanent, the prevalence of the COVID-19 is also high. Figure 1 reveals that the density of infected people living in areas close to metro lines was much higher than the rest of the city. Accordingly, as proven in the previous studies [65–68], in congested places such as business centres and public transportation, a quicker spread of the virus is expected. In agreement with the findings of Pourghasemi et al. [69], it can be concluded that the areas considered public spaces of the city (such as subway stations, parks, commercial areas, and pilgrimage centres) are high-risk areas since adherence to health protocols there has been weaker than in the residential areas. However, we should not forget that the marginal areas (e.g. north-western areas) of the city have less potential accessibility compared with central urban, due to the high density of hospitals and other health care centres [15, 70, 72]. Most of these neighbourhoods are low-income parts of the city and they are very vulnerable to the pandemic.

The downtown of Mashhad is no exception from other areas with a high prevalence of COVID-19 cases, but in line with the points made by Pourghasemi et al. [69], it presents a multitude of places where high transmission of the virus can occur. Because the COVID-19 infection did not necessarily form hotspots only where the population density is high in our study, the results of the current study are neither are in line with the findings of Tang et al. [21], who showed the hotspots where the virus originated in China nor do they correspond to the findings of Fan et al. [22], who found the hotspots to be mainly restricted to densely populated or developed areas. Accordingly, we instead found it useful to follow other patterns and variables in each district of the city, such as the household’s socioeconomic status, urban built environment and air quality index. As the spatiotemporal maps show (Fig. 6), we found the spatiotemporal pattern of the COVID-19 to be very dynamic and unpredictable, which is in agreement with the findings of Arauzo-Carod [25]. Indeed, in agreement with other spatial studies [23, 24] on COVID-19 distribution and trends, we found that the spatial patterns of the infection in the city of Mashhad were clustered rather than random. Identifying such clusters over time would help urban health planners to implement tailored lockdown strategies. For example, HH and HL areas should be lock downed first (Fig. 7).

### Limitations

Only 33% of all included cases were approved by RT-PCR testing and the rest of the cases were clinically confirmed. This is because of the retrospective study design and including a large number of COVID-19 cases. Interestingly, however, the obtained results were in line with other studies. The presence of such data can make the results more generalizable. As previously shown in other studies, hypertension affects the prognosis of the COVID-19 patients negatively, but we had no separate data about it and it was therefore collected under the heading CVD. As well as, the absence of data on patients who were still hospitalized at the time of reporting may have biased the findings. In order to address these types of data deficiencies in the future, using structured forms for clinical data gathering and also developing registry programs are highly recommended [73]. Due to the short period (3 months) covered, the advantages of spatial analysis cannot tell us more now. The spatial and temporal dynamics of the disease need to be studied over a longer period in order to provide more effective solutions. However, due to our discovery of hotspots and coldspots, city policymakers can concentrate their solutions in the central, eastern, and north-eastern parts of the city. In particular, central sectors need more sustainable solutions. Finally, it was possible that some patients had not gone to the hospitals, thus we did not have their data.

## Conclusions

Older adults and people with severe underlying medical conditions are at higher risk for developing complications from COVID-19 infection. By applying spatiotemporal methods to identify the transmission trends and clustering patterns of the disease, we expect the current study to improve our understanding of which factors are of practical importance for transmission of the disease thereby providing documentation that can assist policymakers. In response to the COVID-19 epidemic, along with efforts to find medical solutions, future studies should focus on predicting disease recurrence in cities and places where people tend to aggregate. Medical studies should be conducted in conjunction with socio-economical, spatial, and environmental studies to provide comprehensive and integrated solutions to control and prevent epidemic diseases in general.

## Data Availability

The datasets used and/or analysed during the current study are publicly available from the corresponding authors on request.

## Abbreviations

SARS-CoV-2: Severe Acute Respiratory Syndrome Coronavirus 2
ICU: Intensive Care Units
WHO: World Health Organization
SOFA: Sequential Organ Failure Assessment Score
GISs: Geographic Information Systems
RT-PCR: Reverse Transcription Polymerase Chain Reaction
LOS: Length of Stay
MUMS: Mashhad University of Medical Sciences
IQR: Inter Quartile Range
OR: Odds Ratio
CI: Confidence Interval
KDE: Kernel Density Estimation
GMI: Global Moran’s Index
ALMI: Anselin’s Local Moran’s Index
CVD: Cardiovascular Diseases
CRD: Chronic Renal Diseases
COPD: Chronic Obstructive Pulmonary Diseases
CBD: Central Business District
HH: High-High
HL: High-Low
LL: Low-Low
LH: Low-High
CDC: Centre for Disease Control and Prevention
CFR: Case Fatality Rate
